# Photosynthetic and Photoprotective Responses to Steady-State and Fluctuating Light in the Shade-Demanding Crop *Amorphophallus xiei* Grown in Intercropping and Monoculture Systems

**DOI:** 10.3389/fpls.2021.663473

**Published:** 2021-05-21

**Authors:** Jinyan Zhang, Shengpu Shuang, Ling Zhang, Shiqing Xie, Junwen Chen

**Affiliations:** ^1^College of Agronomy and Biotechnology, Yunnan Agricultural University, Kunming, China; ^2^Key Laboratory of Medicinal Plant Biology of Yunnan Province, Yunnan Agricultural University, Kunming, China; ^3^National and Local Joint Engineering Research Center on Germplasm Innovation and Utilization of Chinese Medicinal Materials in Southwestern China, Yunnan Agricultural University, Kunming, China

**Keywords:** monoculture, intercropping, antioxidant defense, photoprotection, sunflecks, *Amorphophallus xiei*

## Abstract

Photosynthetic and photoprotective responses to simulated sunflecks were examined in the shade-demanding crop *Amorphophallus xiei* intercropped with maize (intercropping condition) or grown in an adjacent open site (monoculture condition). Both intercropping leaves and monoculture leaves exhibited very fast induction responses. The times taken to achieve 90% maximum net photosynthetic rate in intercropping leaves and monoculture leaves were 198.3 ± 27.4 s and 223.7 ± 20.5 s during the photosynthetic induction, respectively. During an 8-min simulated sunfleck, the proportion of excess excited energy dissipated through the xanthophyll cycle-dependent pathway (*Φ*_NPQ_) and dissipated through constitutive thermal dissipation and the fluorescence (*Φ*_f, d_) pathway increased quickly to its maximum, and then plateaued slowly to a steady state in both intercropping and monoculture leaves. When the illumination was gradually increased within photosystem II (PSII), *Φ*_NPQ_ increased quicker and to a higher level in monoculture leaves than in intercropping leaves. Relative to their monoculture counterparts, intercropping leaves exhibited a significantly lower accumulation of oxygen free radicals, a significantly higher content of chlorophyll, and a similar content of malondialdehyde. Although monoculture leaves exhibited a larger mass-based pool size of xanthophyll cycle [V (violaxanthin) + A (antheraxanthin) + Z (zeaxanthin)] than intercropping leaves, intercropping leaves had a higher ratio of (Z + A)/(V + Z + A) than monoculture leaves. intercropping leaves had markedly higher glutathione content and ascorbate-peroxidase activity than their monoculture counterparts. Similar activities of catalase, peroxidase, dehydroascorbate reductase, and monodehydroascorbate were found in both systems. Only superoxide dismutase activity and ascorbate content were lower in the intercropping leaves than in their monoculture counterparts. Overall, the xanthophyll cycle-dependent energy dissipation and the enzymatic antioxidant defense system are important for protecting plants from photooxidation in an intercropping system with intense sunflecks.

## Introduction

Crop production is a complex light-driven system. Around 90% of the dry matter accumulated in crops comes from photosynthetic production (Li et al., 2013), and thus photosynthesis is one of the most important factors determining yield in crops. Light is essential for photosynthesis in plants, however, it is a highly heterogeneous resource in nature, and its intensity (irradiance) drastically changes with time and space. Plants experience dynamic irradiance fluctuations, even when growing in open habitats. Understory plants experience extreme fluctuations in light conditions, owing to the leaves and stems of other plants growing above them (Vierling and Wessman, 2000; Huber et al., 2020; Yamori et al., 2020). The light environment inhabited by understory plants is characterized by low-intensity diffuse irradiance punctuated by intense sunflecks that may last from seconds to minutes (Tausz et al., 2005; Alter et al., 2012). It has been estimated that sunflecks may account for 20–80% of the total irradiance (Watling et al., 1997; Augspurger et al., 2005) and result in 30–60% of total daily carbon gain for understory plants (Chazdon et al., 1988; Leakey et al., 2005). Thus, the use of sunflecks is of paramount importance for the overall assimilation balance of understory plants. Over the past few decades, numerous studies have examined the photosynthetic responses of understory plants to dynamic light (Kaiser et al., 2015). However, the photosynthetic responses of the crop plant to dynamic light sunflecks have been largely ignored.

Intercropping system involved growing two or more crops simultaneously and mixed together in the same field (Ofori and Stern, 1987). Under an intercropping system, the light environment inhabited by intercropping crops is also very similar to that inhabited by understory plants. The objective of intercropping is to obtain a greater yield on a given piece of land, by high-efficiency use of resources that would otherwise not be used by a single crop (Evans et al., 2001; George and Jeruto, 2010). Until now, most studies on the intercropping model have focused on total yield and economic benefits of crops (Ghosh, 2004; Ghosh et al., 2006a, b), crop inter-specific interactions in an intercropping system (Zhang et al., 2008; Gao et al., 2009; Li et al., 2014), and pest and disease management in an intercropping system (Zhu et al., 2000). Furthermore, the light harvested by the bottom crop is highly dynamic, both temporally and spatially, depending on the height of the upper crop in intercropping system and multiple factors such as solar angle, cloud cover and wind speed (Yao et al., 2017). Previous studies have shown that intercropping maize (*Zea mays*) with soybean (*Glycine max*) can reduce the incidence of leaf blight and rust and reduce the density of weeds (Iqbal et al., 2018; Nourbakhsh et al., 2019). The distribution of light in the intercropping system was also affected by the shading of the higher crop maize (Liu et al., 2017, 2018). However, little attention has been paid to photosynthetic responses of the bottom crop to sunflecks in intercropping system.

Sunflecks are potentially harmful to plants, because the time-lag between the onset of sunflecks and the achievement of maximum photosynthetic rate may cause a temporary excess in the light energy absorbed by a leaf during a sunfleck, above what is needed for photosynthesis (Watling et al., 1997; Thiele et al., 1998; Tausz et al., 2005). Thus, intense sunflecks may result in photoinhibition and even oxidative damage to photosynthetic apparatus. However, plants respond to these daily and have evolved a variety of mechanisms to avoid severe photoinhibitory and oxidative damage (Morosinotto et al., 2003; Rentel and Knight, 2004; Förster et al., 2011; Belgio et al., 2018). To protect themselves from toxic oxygen intermediates, plants employ a highly intricate defense system, which includes enzymatic and non-enzymatic antioxidant systems for scavenging reactive oxygen species (ROS) (Sundar et al., 2004). Additionally, xanthophyll cycle-dependent heat dissipation is considered to be a highly effective photoprotective mechanism that helps to minimize oxidative damage to photosynthetic apparatus (Lavaud, 2007; Goss and Jakob, 2010; Zhang et al., 2012). Previous efforts have primarily focused on the photoprotective mechanisms of plants under steady-state high-light condition (Logan et al., 1997; Watling et al., 1997; Tausz et al., 2005; Demmig-Adams and Adams, 2006; Förster et al., 2011; Belgio et al., 2018), with little focus on the photoprotective mechanisms of plants under dynamic high-light condition, especially the photosynthetic and photosynthetic induction characteristics of dwarf crops under intercropping condition. Little is also known about the adaptation of crops to light environmentin an intercropping system.

In the present study, our targeted species was *Amorphophallus* sp., a perennial herbaceous plant, which belongs to the genus *Amorphophallus* Blum of the family Araceae and is also a typical shade-demanding crop (Liu, 2004). As a food source, *Amorphophallus konjac* has long been used in China, Japan, and South East Asia (Chua et al., 2010) and the food made from the root of this plant is widely known in English by its Japanese name, konjac. *Amorphophallus xiei* originated from the deeply shaded understory of the tropical forest (Li and Dao, 2006), and gradually became one of the dominant cultivated species of konjac in Yunnan Province, China. Maize is a sun-demanding and high stalk crop. Intercropping between *A. xiei* and maize allows both crops to make full use of light sources at different levels of space, and this intercropping is a common mode of cultivation in production. In view of the overriding importance of sunflecks in understory habitats, we expected that *A. xiei* would have a very fast response to sunflecks when they were intercropped with maize. Meanwhile, we also predicted that the intercropping crop *A. xiei* would have a robust photoprotective mechanism, such as xanthophyll cycle-dependent energy dissipation and an antioxidant defense system that is comparable to that of their monoculture counterparts. Therefore, the species might efficiently protect itself from photo-oxidative stress when subject to high-intensity sunflecks.

## Materials and Methods

### Study Site and Plant Material

Field experiments were carried out at an experimental farm at the Dehong Agricultural Technology Extension Center, which is located in Mangshi (98°01′–98°44′ E and 24°05′–24°39′ N), Yunnan Province, China. The annual average precipitation and temperature at the study site is 1654.6 mm and 19.6°C, respectively. The rainy season is from May to October, when the total precipitation accounts for 90% of annual precipitation. The dry season is from November to April.

*A. xiei* was first found in the understory of a tropical forest in the southwest of Yunnan Province, China, more than 14 years ago (Li and Dao, 2006). Currently, this crop is widely cultivated by local farmers. *A. xiei* is a typical shade-demanding crop (Han et al., 2013), and thus this crop is grown under a shade growth house or in an intercropping system. *A. xiei* is also infrequently grown under a monoculture model, but it does not grow well and has a reduced yield of underground bulbs.

### Experimental Design

The variety of maize used in the present experiment is Yunrui 8, a variety widely cultivated in Yunnan Province, China, and commonly used in intercropping system with *A. xiei*. The experimental bulbs of *A. xiei* were provided by the Extension Center of Agricultural Technology of the Dehong State and weighed 120–150 g. Maize seeds were purchased from local seed companies. Bulbs were sown before the onset of the rainy season. After the leaves of *A. xiei* emerged, the maize seedlings were transported to the intercropping field. As the maize seedling grew, they began to shade the individuals of *A. xiei*. In the monoculture system, each plot contained 10 rows of *A. xiei*. In the intercropping system, each plot contained four rows of konjac and the six rows of maize. There were ten plots for both the monoculture system and intercropping system. The plots were arranged in a completely randomized experimental design. All plots were fertilized with 150 kg N ha^–1^ (urea), 120 kg P_2_O_5_ ha^–1^ (superphosphate), 100 kg K_2_O ha^–1^ (potassium chloride), and 20,000 kg organic fertilizer ha^–1^. Besides the N fertilizer, the remaining was used as a base fertilizer. Thirty percent of N was used as a base fertilizer, and 70% of N as topdressing that was applied 50 days after seedling transplantation. All plots were watered by an automatic irrigation system. The crops were well protected from diseases, pests, and weeds. Field measurements were conducted in the middle of August when *A. xiei* was growing vigorously. Leaf discs was punched, submerged into liquid nitrogen, and transported to the lab for biochemical analysis.

### Specific Leaf Area

The tagged leaves were cut down, and the area of these leaves was determined using a leaf area meter (Li-Cor, Lincoln, NE, United States). Afterward, the leaves were oven dried to constant weight. Specific leaf area (SLA) was calculated as the ratio of leaf area to leaf dry weight (g cm^–2^).

### Steady-State Gas Exchange

Light-response curves and CO_2_-response curves were completed between 09:30 and 11:30 using a portable photosynthesis apparatus (LI-6400XT). The tagged leaves were enclosed in a chamber where light was controlled at 800 μmol m^–2^ s^–1^ by a built-in LED light source, temperature at 25°C by setting block temperature, and CO_2_ concentration at 380 μmol mol^–1^ by a CO_2_ injection system. During the measurements of light-response curves, temperature and CO_2_ concentration in the chamber was retained at 25°C and 380 μmol mol^–1^, respectively, but light intensity was sequentially reduced in the following order: 1,500, 1,000, 800, 600, 400, 300, 200, 150, 100, 80, 60, 40, 20, and 0 μmol m^–2^ s^–1^. To complete the measurements, an automatic measurement program of light-response curves was conducted. The relationship between net photosynthetic assimilation (*P*_n_) and photon flux density (PFD) was fitted to a non-rectangular hyperbola (Webb et al., 1974): *P*_n_ (PFD) = *P*_max_-c × e^–b*PFD^, where *P*_max_ is the maximum net photosynthetic assimilation under saturating light. The light saturating point (LSP) was estimated from ln (0.1 × a/c) × (−1/b). The apparent quantum yield (AQY) and light compensation point (LCP) were estimated as the slope and x-intercept of the linear region of the light response curve, respectively.

The relationship between *P*_n_ and intercellular CO_2_ concentration (*C*_i_) was evaluated by a CO_2_ response curve. The tagged leaves were enclosed in a chamber where temperature, PFD, and CO_2_ concentration were adjusted to 25°C, 800 μmol m^–2^ s^–1^, and 400μmol mol^–1^, respectively, and the leaf was fully induced by illumination for more than 15 min. Afterward, an automatic program of CO_2_ response curves was conducted to measure the change in gas exchange rate with varied CO_2_ concentration. The CO_2_ concentration in the chamber was varied in the following order: 400, 300, 200, 150, 100, 50, 400, 600, 800, 1,000, and 1,200 μmol mol^–1^. CO_2_ response curves were obtained by fitting the data to a non-rectangular hyperbola. Carboxylation efficiency (CE) was estimated by the slope of the linear region of the CO_2_ response curve.

### Photosynthetic Induction During Simulated Sunflecks

The tagged leaves were dark-adapted overnight by covering the canopy with a black plastic bag. In the morning of the following day, the fully dark-adapted leaves were enclosed in a dark chamber where PPFD was 20 μmol m^–2^ s^–1^. When the readings were completely stable, gas exchange rate was then automatically recorded at 30-s intervals. Afterward, PPFD was increased by one step to a high light level of 1,500 μmol m^–2^ s^–1^, and gas exchange rate was automatically recorded at 30-s intervals until stable readings were achieved. The process of photosynthetic induction was completed through an automatic program of time-lamp responses. The temporal change in *P*_net_ during photosynthetic induction was fitted to a non-rectangular hyperbola (Tausz et al., 2005):

P(t)n=P+shade(P-maxP)shade×(1--e)-t/t1,

where *P*_shade_ is the initial photosynthetic assimilation in the dark, *P*_max_ is the maximum *P*_net_ during the simulated sunfleck, t is the time from the onset of the simulated sunfleck, and t_1_ is a characteristic time constant. The times to reach 50 and 90% of *A*_max_ (*T*_50__%_ and *T*_90__%_) were calculated from the photosynthetic induction curve.

### Chlorophyll Fluorescence

At predawn, minimum and maximum chlorophyll (Chl) *a* fluorescence yields (*F*_0_ and *F*_m_) were measured in the fully dark-adapted leaves, and the maximum efficiency of PSII photochemistry (*F*_v_/*F*_m_) was calculated as (*F*_m_–*F*_0_)/*F*_m_. Minimum, variable, maximum, and steady-state fluorescence intensity (*F*_0_′, *F*_v_′, *F*_m_′, and *F*_s_) were recorded in measurements of light response curves and photosynthetic induction curves. Maximum efficiency of PSII photochemistry in the light (*F*_v_′/*F*_m_′) was calculated as (*F*_m_′–*F*_0_′)/*F*_m_′, actual efficiency of PSII photochemistry in the light (Δ*F*/*F*_m_′) as (*F*_m_′–*F*_s_)/*F*_m_′, electron transport rate (ETR) as Δ*F*/*F*_m_′ × PFD × 0.84 × 0.5, non-photochemical quenching (NPQ) as (*F*_m_–*F*_m_′)/*F*_m_′, and photochemical quenching (qP) as (*F*_m_′–*F*_s_)/(*F*_m_′–*F*_0_′).

The fate of absorbed light energy by PSII was estimated by the method described by Hendrickson et al. (2004). In brief, the absorbed light energy was consumed by three competitive pathways: (1) Φ_f__,d_ = *F*_s_/*F*_m_, (2) Φ_PSII_ = (*F*_m_′–*F*_s_)/*F*_m_′, and (3) Φ_NPQ_ = *F*_s_/*F*_m_′–*F*_s_/*F*_m_. Φ_f, d_ is the sum of the fractions of light energy absorbed by PSII that is lost by either constitutive thermal dissipation or via fluorescence, Φ_PSII_ corresponds to the fraction of absorbed light energy that is consumed by photosynthetic electron transport, and Φ_NPQ_ is the fraction of absorbed light energy that is dissipated thermally via light-dependent non-photochemical quenching processes. The allocation of absorbed light energy by PSII was analyzed in the fully light-induced leaves that were used to measure light response curves, and in the thoroughly dark-adapted leaves that were used to measure photosynthetic induction curves.

### Photosynthetic Pigment Determination

At midday on a clear day in August, leaf discs (2 cm in diameter) were punched from the tagged plants, and were then immediately submerged in liquid nitrogen. The samplings were taken to the lab for biochemical analysis. Carotenoid and Chl content was determined according to the method described by Förster et al. (2009). In brief, pigments were first extracted from individual discs in a microfuge tube with 0.6 mL of ethyl acetate: acetone (60:40, v/v) and shaken at 30 Hz for 2 min with a stainless steel ball (2-mm diameter), 0.5 mL of water was then added, followed by 5-min centrifugation at 13,000 rpm. The pigment containing the upper layer was transferred to a fresh microfuge tube, centrifuged as before, and 0.1 mL of the pigment solution was placed in vials for high-performance liquid chromatography analysis. The analysis was conducted on a Agilent 1200 fitted with a Waters Spherosorb ODS2 column, using a linear gradient from 100 to 33% acetonitrile: water (90:10[v/v] with 0.1% triethanolamine) into ethyl acetate over 60 min. Pigments were identified by retention times and spectra, and carotenoid concentrations were calculated using conversion factors for A_440_, obtained from pure pigments.

### Antioxidant Metabolite

Reduced ascorbate was determined as described by Arakawa et al. (1981). Ascorbate was extracted from 0.2 g fresh leaf tissue with 5 mL 5% (w/v) trichloroacetic acid (TCA). In brief, ascorbate was measured in the supernatant after centrifugation at 20,000 *g* for 10 min using an analytical method based on the reduction of ferric ion to ferrous ion. Reduced glutathione was assessed by dithio-bis-nitrobenzoic acid (DTNB) colorimetry at an absorbance of 412 nm as described by Doulis et al. (1997). Glutathione was extracted from 0.5 g leaf fresh material with 5 mL ice-cold 5% (w/v) TCA containing 5 mM Na_2_-EDTA, followed by centrifugation at 12,000 *g* for 20 min. Afterward, 0.5 mL phosphate buffer (pH 6.8, 0.1 M) and 0.5 mL DTNB (4 mM) were added into 1 mL supernatant, respectively.

### Antioxidant Enzymes

Leaves harvested at midday on a clear day were immediately frozen in liquid nitrogen. Frozen leaves (ca. 0.5 *g*) were crushed into fine powder with a mortar and pestle under liquid nitrogen. Homogenates for antioxidant enzyme analyses were prepared according to the method described by Chen X. Y. et al. (2011). Total superoxide dismutase (SOD) activity was measured according to the method of Giannopolitis and Reis (1977) with some modifications. Each 3-mL reaction mixture contained 50 mM potassium phosphate buffer (pH 7.8), 13 mM methionine, 75 μM nitroblue tetrazolium, 2 μM riboflavin, 10 μM Na_4_-EDTA, and 200 μL enzyme extract. The reaction mixtures were illuminated for 15 min at a light intensity of 4,000 lx. One unit of SOD activity was defined as the amount of the enzyme required to induce 50% inhibition of nitroblue tetrazolium, as monitored at 560 nm.

Activity of catalase (CAT), which decomposes H_2_O_2_, was determined by monitoring the decrease in absorbance at 240 nm (Aebi, 1984). Each 3-mL reaction mixture contained 50 mM potassium phosphate buffer (pH 7.0), 10 mM H_2_O_2_, and 50 μL enzyme extract. One unit of CAT activity was expressed as μmol H_2_O_2_ min^–1^ g^–1^ fresh weight leaves.

Peroxidase (POD) activity was assayed by monitoring the increase in absorbance at 470 nm caused by guaiacol oxidation. Each 3-mL reaction mixture contained 50 mM phosphate buffer (pH 7.0), 16 mM guaiacol, 10 mM H_2_O_2_, and 200 μL enzyme extract. One unit of POD activity was expressed as μmol guaiacol oxidation min^–1^ g^–1^ fresh weight leaves.

Ascorbate peroxidase (APX) activity was analyzed according to the method described by Arakawa et al. (1981). Each 3-mL reaction mixture contained 50 mM potassium phosphate buffer (pH 7.0), 0.1 mM MEDTA-Na_2_, 0.3 mM ascorbic acid, 60 μM H_2_O_2_, and 200 μL enzyme extract. Enzyme activity was determined by monitoring the decrease in absorbance at 290 nm. One unit of APX activity was expressed as μmol ascorbate oxidized min^–1^ g^–1^ fresh weight leaves.

Glutathione reductase (GR) activity was determined by monitoring the disappearance of NADPH at 340 nm. Each 3-mL reaction mixture contained 100 mM potassium phosphate buffer (pH 7.8), 2 mM EDTA, 0.2 mM NADPH, 0.5 mM oxidized glutathione, and 200 μL enzyme extract. One unit of GR activity was expressed as μmol NADPH oxidation min^–1^ g^–1^ fresh weight leaves.

Monodehydroascorbate (MDHAR) activity was measured by monitoring decreases in absorbance at 340 nm, which mirrored the oxidation of NADPH. Each 3-mL reaction mixture contained 25 mM potassium phosphate (pH 7.8), 0.2 mM EDTA, 0.1 mM ascorbate, 0.5 U of ascorbate oxidase, 0.1 mM NADPH, and 100 μL enzyme extract. One unit of MDHAR activity was expressed as μmol NADH min^–1^ g^–1^ fresh weight leaves.

Dehydroascorbate reductase (DHAR) activity was measured by monitoring the increase in absorbance at 265 nm due to the formation of ascorbate. Each 3-mL reaction mixture contained 100 mM potassium phosphate (pH 7.0), 50 mM reduced glutathione, 5 mM dehydroascorbate acid, and 100 μL enzyme extract. One unit of DHAR activity was expressed as μmol reduced ascorbate min^–1^ g^–1^ fresh weight leaves.

### Reactive Oxygen Species

Superoxide ions (O_2_^–^) were measured according to the method of Elstner and Heupel (1976), by monitoring nitrate formation from hydroxylamine in the presence of O_2_. Fresh leaf samples (1.0 g) were ground with liquid nitrogen, homogenized in 5 mL of 50 mM phosphate buffer (pH 7.8), and centrifuged at 12,000 *g* for 10 min at 4°C. The resulting supernatant was then re-centrifuged at 18,000 *g* for 20 min at 4°C. Reaction mixtures consisting of 1 mL supernatant and 1 mL of 1 mM hydroxylamine hydrochloride (using 50 mM phosphate buffer [pH 7.8] as solvent) were incubated at 25°C for 1 h. After addition of 1 mL of 17 mM aniline sulfonic acid and 1 mL of 7 mM α-naphthylamine, the reaction was performed at 25°C for 20 min. Absorbance was recorded at 530 nm using sodium nitrite as a standard.

### Statistics and Analysis

All the analyses were conducted using the SPSS software package (Chicago, IL, United States), and the variables were given as the mean ± SD. Data were subjected to analyses of variance based on a completely randomized experimental design, and *t*-tests were then used for testing significant differences between measurements of intercropping and monoculture *A. xiei*. In addition, only *P* < 0.05 were accepted as significant.

## Results

### Determination of Intercropping Pattern and Light Transmission in the Intercropping System

**FIGURE 1:**
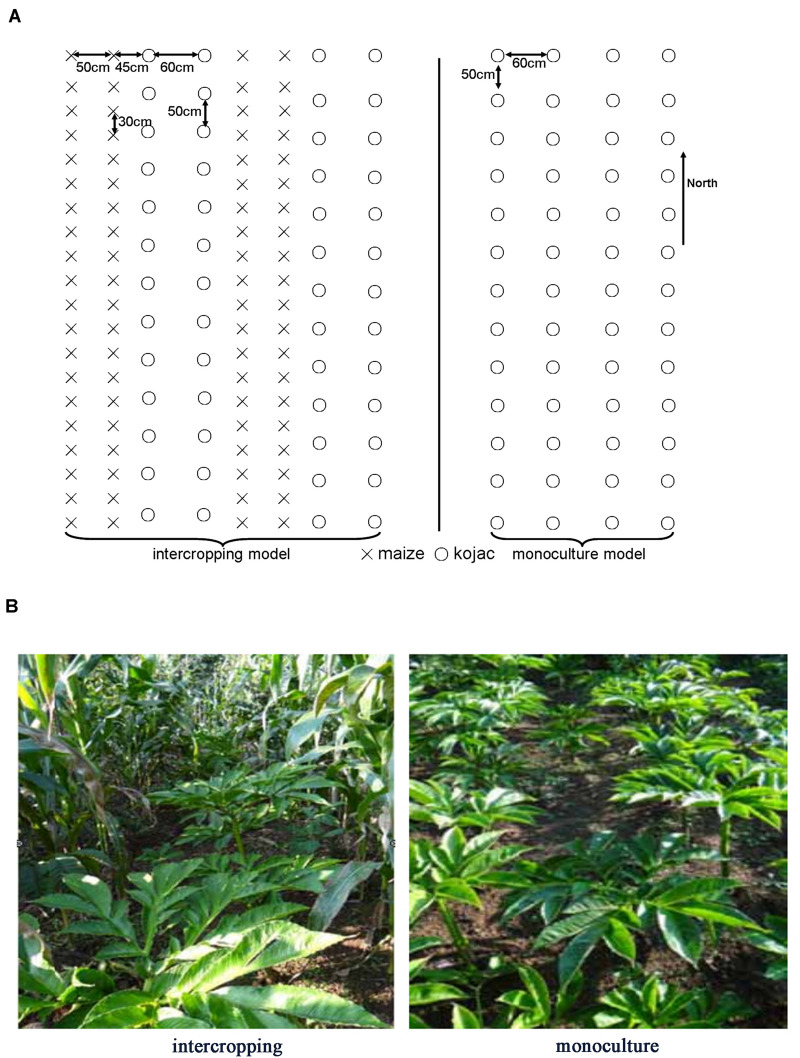
**(A)** Experimental design for the intercropping and monoculture of *Amorphophallus xiei*. In the monoculture system, the distance between rows and plants was 60 and 50 cm, respectively. In the intercropping system, two rows of *A. xiei* were intercropped with two rows of maize. The row and plant distance for *A. xiei* under the intercropping system was the same as in the monoculture system. In the intercropping system, the distance between rows of *A. xiei* and rows of maize was 45 cm, and the row and plant distance for maize was 50 and 30 cm, respectively. The circles represent the *A. xiei*, crosses represent the maize. **(B)** The landscape of shade-demanding crop *Amorphophallus xiei* grown in intercropping and monoculture systems. The two crops involved in the intercropping system are konjac and maize.

**FIGURE 2:**
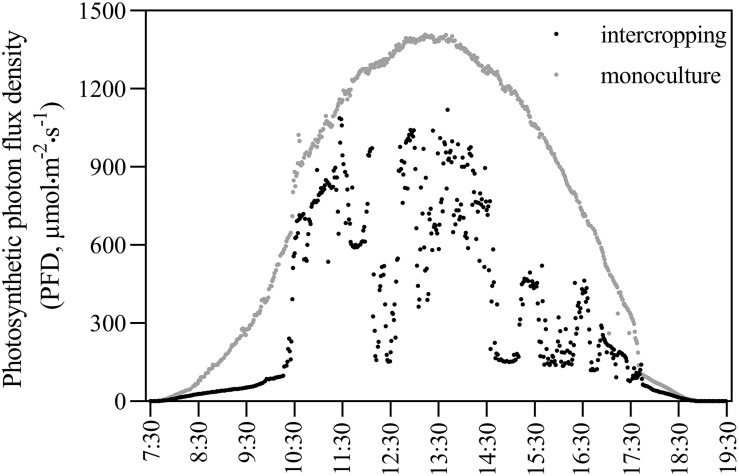
Diurnal variation of photosynthetic photon flux density in intercropping and monoculture systems.

In the monoculture system, the distance between rows and plants was 60 cm and 50 cm, respectively. In the intercropping system, two rows of *A. xiei* were intercropped with two rows of maize. The row and plant distance for *A. xiei* under the intercropping system was the same as in the monoculture system. In the intercropping system, the distance between rows of *A. xiei* and rows of maize was 45 cm, and the row and plant distance for maize was 50 and 30 cm, respectively. The design of the plane graph is shown as in Figure 1. On a sunny day, the light intensity of intercropping and monoculture systems were collected every 10 s between 7:30 and 19:30 using a Li-1500 (Li-Cor, United States) light quantum meter, the measurements were taken on the very same day. The intensity of full sunlight (FL) was also measured as a control (monoculture systems) (Figure 2). The percentage of light intensity in the intercropping to the full sunlight was used as an indicator of the light intensity of *A. xiei* growth light environment. The light transmission in the intercropping was measured to be around 45%FL (Figure 2).

### Photosynthetic Pigments, Steady-State Photosynthetic Characteristics Under the Intercropping System

**FIGURE 3:**
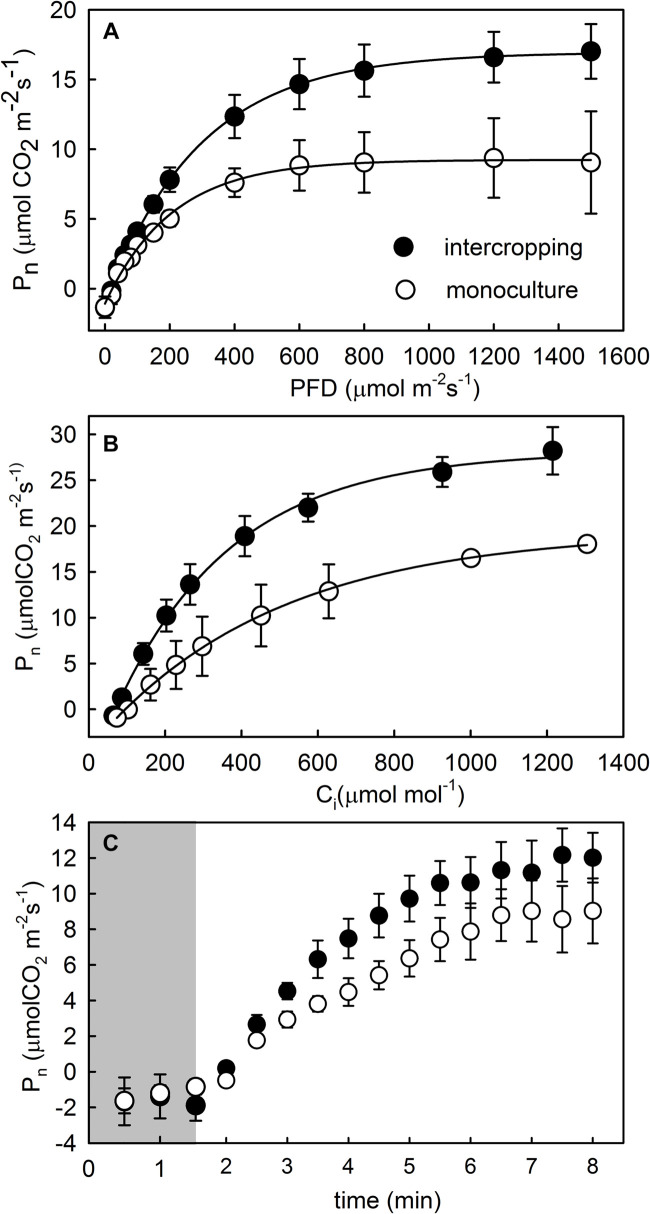
Photosynthetic light response **(A)**, CO_2_ response **(B)**, and photosynthetic induction **(C)** curves of fully mature leaves of *morphophallus xiei* under different cropping system. Each point is the mean ± SD of five replicates. *P*_n_, net photosynthesis; PFD, photo flux density; C_i_, intercellular CO_2_ concentration; closed parts of photosynthetic induction curves indicate a response to low light, and open parts indicate a response to high light.

**FIGURE 4:**
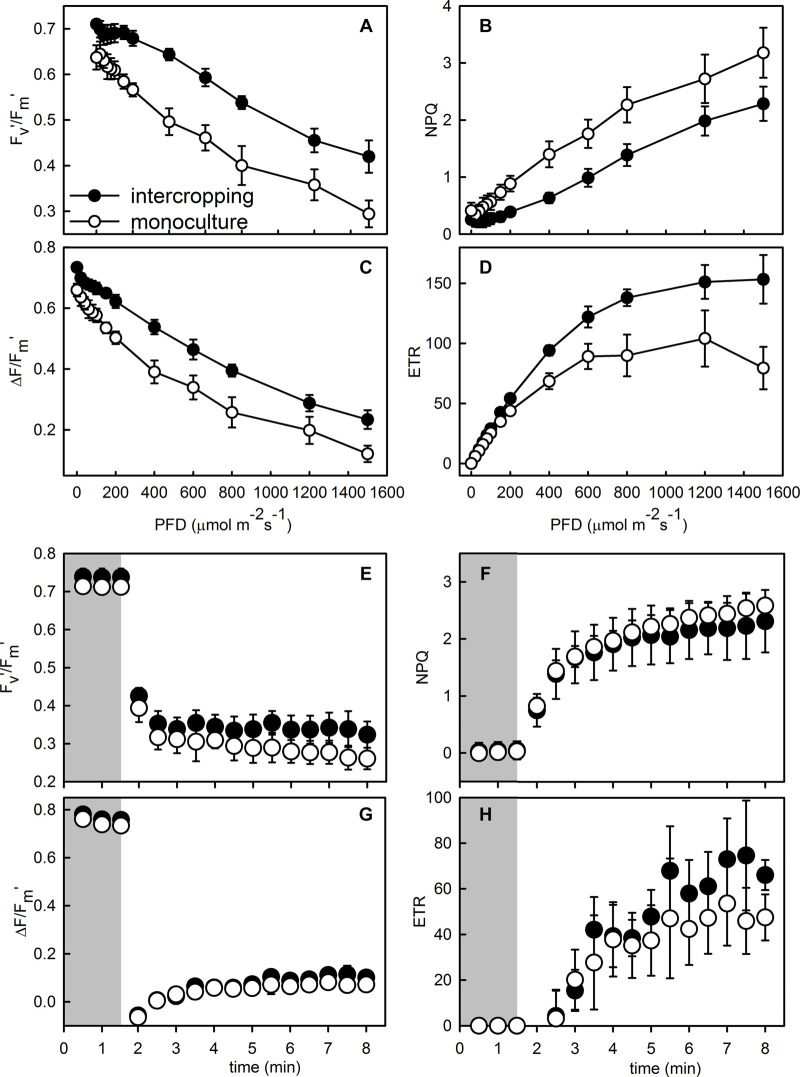
Responses of maximum photochemical efficiency of PSII in the light (*F*_v_′/*F*_m_′), actual photochemical efficiency of PSII (Δ*F*/*F*_m_′), non-photochemical quenching (NPQ), and electron transport rate (ETR) to photo flux density **(A–D)** and the simulated sunfleck **(E–H)** in the leaves of *morphophallus xiei* grown under different cropping system. Each point is the mean ± SD of five replicates. Closed parts of responses to the simulated sunfleck indicate a response to low light, and open parts indicate a response to high light.

The contents of area-based Chla, Chlb, and Chl_a__+__b_ were significantly higher in intercropping leaves than in monoculture leaves of *A. xiei* (Table 1). The contents of mass-based Chla, Chlb, and Chl_a__+__b_ showed the same pattern as shown in area-based expression (Table 1). The steady-state response of net CO_2_ exchange to PFD in intercropping leaves and monoculture leaves showed an obvious different pattern (Figure 3A), and such responses implied that *A. xiei* was also a typically shade-demanding crop. The maximum net photosynthetic rate (*P*_max_) in intercropping leaves and monoculture leaves was 16.97 μmol m^–2^ s^–1^ and 9.46 μmol m^–2^ s^–1^, respectively, the former being significantly higher than the latter (*P* ≤ 0.01; Table 1). The apparent quantum yield (AQY) of intercropping leaves was 0.06 ± 0.01 mol CO_2_ mo1^–1^ photons, significantly higher than that of monoculture leaves (Table 1). The apparent carboxylation efficiency (CE) of understory leaves was 0.10 ± 0.003 mol CO_2_ m^–2^ s^–1^, also significantly higher than that of monoculture leaves. However, it was very interesting that no significant difference was found between intercropping leaves and monoculture leaves in the dark respiration rate (*R*_d_), light compensation point (LCP), light saturation point (LSP) (Table 1). Under identical PFD, higher values of maximum efficiency of PSII photochemistry in the light (*F*_v_′/*F*_m_′), actual efficiency of PSII photochemistry in the light (Δ*F*/*F*_m_′) and electron transport rate (ETR) were recorded in the intercropping leaves than in the monoculture leaves; however, a reverse trend was observed for non-photochemical quenching (NPQ) (Figures 4A–D).

**TABLE 1:** Photosynthetic-related characteristics in the leaves of *A. xiei* under different cropping systems (Data are means ± SD, *n* = 5–10).

**Variables**	**Treatment**		***F-*values**
	**Monoculture**	**Intercropping**	
Area-based Chla (mg cm^–2^)	4.750.24b	7.350.28a	22.47***
Area-based Chlb (mg cm^–2^)	0.710.04b	1.050.03a	20.05***
Area-based Chla + b (mg cm^–2^)	5.460.27b	8.400.32a	22.18***
Mass-based Chla (mg g^–1^)	196.779.84b	294.8711.19a	20.82***
Mass-based Chlb (mg g^–1^)	29.561.52b	42.021.52a	18.34***
Mass-based Chla + b (mg g^–1^)	226.3311.35b	336.8912.71a	20.52***
*P*_max_ (μmol m^–2^ s^–1^)	9.462.9b	16.972.11a	4.52**
*R*_d_ (μmol m^–2^ s^–1^)	1.060.38	1.190.35	0.56
LCP (μmol m^–2^ s^–1^)	22.8210.96	19.795.7	0.54
LSP (μmol m^–2^ s^–1^)	515.5212.2	691.376.1	1.74
CE (μmol mol^–1^)	0.050.02b	0.10.003a	7.55***
AQY	0.050.01	0.060.01	2.9
*T*_50__%_ (s)	117.017.6	83.222.6a	2.24
*T*_90__%_ (s)	223.720.5	198.327.4	1.42
SLA (cm^2^ g^–1^)	249.327.6b	289.523.5a	3.41**
*F*_v_/*F*_m__–__predawn_	0.620.01b	0.780.02a	20.53***

Different letters in the same row indicate statistically significant differences at the level of P ≤ 0.05. *P < 0.05, **P < 0.01, and ***P < 0.001. Chl-a, chlorophyll a; Chl-b, chlorophyll b; Chl, chlorophyll, P_*max*_, maximum net photosynthetic rate; R_*d*_, dark respiration rate; LCP, light compensation point; LSP, light saturation point; CE, apparent carboxylation efficiency; AQY, apparent quantum yield; T_50__%_ and T_90__%_, time reach to 50 and 90% of P_*max*_ during the photosynthetic induction; SLA, specific leaf area; F_*v*_/F_*m*__–__*predawn*_, maximal photochemical efficiency of PSII at predawn.

### Photosynthetic Induction Characteristics Under the Intercropping System

When the PFD was increased from 20 to 1,500 μmol m^–2^ s^–1^, the time course of photosynthetic induction in both intercropping leaves and monoculture leaves resembled a hyperbolic response in *P*_n__et_. The hyperbolic response was characterized by a rapid increase in *P*_n__et_ to 50–90% (Figure 3C). The time to achieve maximum gas exchange rate appeared to be similar between intercropping leaves and monoculture leaves during the simulated sunflecks (Figures 4E–H). Moreover, no significant statistical difference was found in the values of dynamic photosynthetic induction-related traits such as *T*_50__%_ and *T*_90__%_ between intercropping leaves and monoculture leaves, whereas intercropping leaves had lower *T*_50__%_ and *T*_90__%_ than monoculture leaves (Table 1).

**FIGURE 5:**
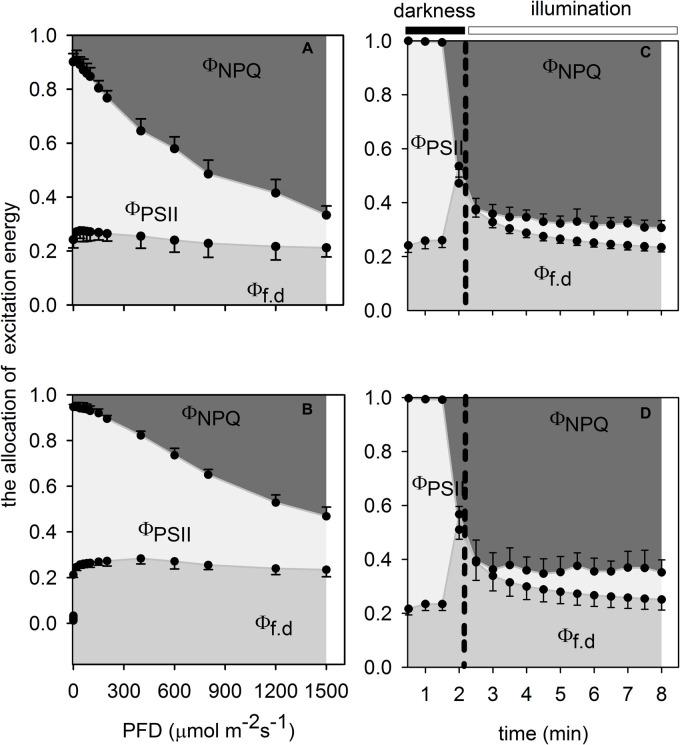
Effects of photon flux density **(A,B)** and the simulated sunfleck **(C,D)** on the energy allocation pattern in the leaves of *A. xiei* under different cropping system: monoculture **(A,C)** and intercropping **(B,D)**. Φ_NPQ_: the fraction of absorbed light energy lost to light-dependent thermal dissipation, Φ_PSII_: the fraction of absorbed light energy lost to photochemistry of PSII, and Φ_f__,d_: the fraction of absorbed light energy lost to fluorescence and constitutive thermal dissipation. Each point is the mean ± SD of five replicates.

Monoculture leaves had lower maximal photochemical efficiency of PSII at predawn (*F*_v_/*F*_m__–__predawn_) than that of understory leaves (Table 1), indicating that the former suffered photoinhibition to a certain extent. Both monoculture leaves and intercropping leaves generally showed actual photochemical efficiency (Φ_PSII_) to decrease rapidly to the minimum value and increase slowly thereafter to the steady-state but the proportion of excess excited energy dissipated through xanthophyll cycle-dependent pathway (Φ_NPQ_) and the proportion of excess excited energy dissipated through constitutive thermal dissipation and fluorescence (Φ_f, d_) to increase fast to the maximum value and then decline slowly to the steady-state (Figures 5A,B). However, the intercropping leaves allocated more absorbed light energy to Φ_PSII_ than the monoculture leaves which in turn partitioned more light absorbed light energy to Φ_NPQ_ during photosynthetic induction (Figures 5C,D). When intercropping leaves and monoculture leaves were exposed to a sudden increase in PFD from 20 to 1,500 μmol m^–2^ s^–1^ during the simulated sunfleck, the non-photochemical quenching (NPQ) and electron transport rates (ETR) increased rapidly at first and then increased slowly to the steady-state (Figures 4F,H); however, the Φ_PSII_ of photosystem II in the light dropped rapidly to the minimum value and increased slowly thereafter to the steady state (Figures 5A,B), and the photochemical efficiency of PSII in the light (*F*_v_′/*F*_m_′) decreased rapidly at first and then dropped slowly to the steady-state (Figure 4E). In the process of photosynthetic induction, the changes of *F*_v_′/*F*_m_′ appeared to decrease more rapidly and largely in the monoculture leaves than in the intercropping leaves; the changes of NPQ appeared to increase more rapidly and largely in the monoculture leaves than in the intercropping leaves, whereas the changes of Φ_PSII_ and ETR appeared to increase more rapidly and largely in the intercropping leaves than in the monoculture leaves (Figures 4, 5).

### Photoprotection and Antioxidant Activity Under the Intercropping System

The allocation pattern of energy in the process of light response in both intercropping leaves and monoculture leaves was shown in Figures 5A,B. The proportion of energy allocated to Φ_NPQ_ gradually increased with an increase in PFD in both monoculture leaves and intercropping leaves, and this proportion was significantly higher in the former than in the latter at the same PPFD. However, the proportion of energy allocated to Φ_f, d_ remained almost constant with an increase in PPFD in the two treated leaves (Figures 5A,B). The xanthophylls were believed to be involved either directly or indirectly in the non-photochemical quenching (NPQ) of excess light energy in the antenna of PSII. There were significant differences in mass-based pool size of xanthophyll cycle i.e., (V + A + Z) between monoculture leaves and intercropping leaves; however, no significant statistical difference was found in area-base pool size of xanthophyll cycle between intercropping leaves and monoculture leaves (Table 2). The extent of the de-epoxidation of the pigment imterconversion within the xanthophyll cycle can be described by the ratio of (Z + A)/(V + Z + A). The (Z + A)/(V + Z + A) ratio was significantly higher in intercropping leaves than in monoculture leaves (Table 2). The content of V and β-carotenoid was also significantly higher in intercropping leaves than in monoculture leaves (Table 2).

**TABLE 2:** Photoprotective-related parameters in the leaves of *A. xiei* under different cropping systems (Data are mean ± SD, *n* = 5–10).

**Variables**	**Treatment**		***F-*values**
	**Monoculture**	**Intercropping**	
Area-based L (mg cm^–2^)	0.640.04b	0.750.03a	7.33***
Area-based N (mg cm^–2^)	0.130.01b	0.160.01a	13.94***
Area-based β-car (mg cm^–2^)	0.430.02b	0.750.03a	28.27***
Area-based V (mg cm^–2^)	0.350.02a	0.310.01b	6.77***
Area-based A (mg cm^–2^)	0.110.01b	0.120.004a	3.18**
Area-based Z (mg cm^–2^)	0.070.03	0.080.03	1.14
Area-based V + A + Z (mg cm^–2^)	0.530.04	0.510.02	1.71
Mass-based L (mg g^–1^)	26.51.59b	29.91.03a	5.75***
Mass-based N (mg g^–1^)	5.160.26b	6.550.25a	12.25***
Mass-based β-car (mg g^–1^)	17.60.89b	30.21.18a	26.73***
Mass-based V (mg g^–1^)	14.60.72a	12.40.43b	8.38***
Mass-based A (mg g^–1^)	4.470.22	4.60.18	1.51
Mass-based Z (mg g^–1^)	2.931.40	3.330.13	0.90
Mass-based V + A + Z (mg g^–1^)	22.01.72a	20.30.74b	2.86
(A + Z)/(V + A + Z)	0.330.04	0.390.02	4.81***

Different letters in the same row indicate statistically significant differences at the level of ≤ 0.05. *P < 0.05, **P < 0.01, and ***P < 0.001. L, lutein; N, neozanthin; β-car, β-carotene; V, violanxanthin; A, antheroxanthin; Z, zeaxanthin; V + A + Z, xanthophyll cycle pool size.

An obvious difference was observed between intercropping leaves and monoculture leaves in the antioxidative enzyme defense system of *A. xiei* (Table 3). The oxygen free radicals were more likely to accumulate in monoculture leaves than in intercropping leaves (Table 3). However, there was no significant difference between intercropping leaves and monoculture leaves in the MDA content. Moreover, no significant difference was found between the two treated leaves in the activities of CAT, POD, DHAR, and MDAR (Table 3). The SOD activity and ascorbate content were significantly higher in monoculture leaves than in intercropping leaves whereas the glutathione content was significantly lower in the former than in the latter (Table 3).

**TABLE 3:** The antioxidant system in the leaves of *A. xiei* under different cropping systems (Data are mean ± SD, *n* = 5–10).

**Variables**	**Treatment**		***F-*values**
	**Monoculture**	**Intercropping**	
Oxygen free radicals (μmol g^–1^ FW)	91.06.14a	53.03.18b	17.35***
MDA (μmol g^–1^ FW)	5.431.55	6.031.94	0.76
CAT (μmol H_2_O_2_ min^–1^g^–1^ FW)	312.9141.7	320.1119.7	0.47
APX (μmol ASC⋅min^–1^g^–1^ FW)	5.132.49	7.451.71	2.21
SOD (U g^–1^ FW)	142.543.0	103.637.3	2.17
POD (U g^–1^ FW)	227.4106.1	208.094.1	0.78
DHAR (μmol ASC min^–1^g^–1^ FW)	1.390.56	1.230.68	0.36
MDAR (μmol NADPH min^–1^g^–1^ FW)	0.270.2	0.190.14	0.98
Ascorbate (mg g^–1^ FW)	1.290.17a	0.830.074b	7.79***
Glutathione (μmol g^–1^ FW)	35.67.5b	52.49.3a	4.47***

Different letters in the same row indicate statistically significant differences at the level of ≤ 0.05. *P < 0.05, **P < 0.01, and ***P < 0.001. MDA, Malondialdehyde; CAT, Catalase; APX, Ascorbate peroxidase); POD, Peroxidase; SOD, Superoxide dismutase; DHAR, Dehydroascorbate reductase; MDAR, Monodehydroascorbate.

## Discussion

In leaves, the light-capturing surface area built by the plant per unit investment of dry mass is a key trait from a physiological, ecological, and biophysical point of view (Milla and Reich, 2007). It represents a potential revenue stream, and is thus analogous to the potential rate of return on the investment of dry mass in terms of light capture (Westoby et al., 2000). Plants try to increase their light interception ability under low-light condition, and hence they generally acclimate to such conditions by an increase in SLA and Chl content (Evans and Porter, 2001). Our data also supported this argument that the intercropping leaves exhibited higher SLA and Chl content than the monoculture leaves (Table 1). Compared with their monoculture counterparts, the intercropping leaves of *A. xiei* apparently retain more potent photosynthetic capacity and might fix CO_2_ at higher rates when subjected to high intensity of light and high concentrations of CO_2_ (Figure 3 and Table 1), which also indicates that *A. xiei* is a shade-demanding crop species.

In view of the relative importance of sunflecks for daily carbon gain, understory plants are expected to effectively use fluctuating light. The effective use of fluctuating light requires rapid photosynthetic induction, because the photosynthetic induction state determines how rapid the leaf assimilates CO_2_ at a given light intensity (Ozturk et al., 2013). Furthermore, many studies have shown that shade-grown species exhibited a faster photosynthetic induction in response to sunflecks, compared with open-grown species (Leakey et al., 2005; Tausz et al., 2005). However, in our study, the time to achieve 50 or 90% of photosynthetic induction did not differ significantly between the monoculture leaves and the intercropping leaves, although the latter had relatively smaller values of *T*_50__%_ and *T*_90__%_ (Table 1). Despite this, the monoculture and intercropping leaves of *A. xiei* both displayed much faster induction in response to sunflecks (Figure 3C and Table 1) when compared with many other shade-tolerant species (Rijkers et al., 2000; Bai et al., 2008; Zhang et al., 2009; Chen J. W. et al., 2011; Zhang et al., 2012; Chen et al., 2014). Indeed, 90% photosynthetic induction in *A. xiei* was achieved within 4 min (Table 1), which is amongst the fastest response times (photosynthetic induction time constants, *T*_90__%_) as reviewed by Naumburg and Ellsworth (2000) for shade-tolerant species (between 3 and 37 min). In our study, the light environment inhabited by *A. xiei* intercropping with maize was also very similar to that inhabited by understory plants, and in such light environments, sunflecks provide a significant light resource. Thus, for the shade-demanding crop *A. xiei*, a rapid photosynthetic response to sunflecks might be of great importance to its carbon balance, allowing it to maximize the use of light energy from sunflecks and maintain a positive carbon balance, and is thus highly advantageous to its final yield under shade condition of the intercropping system.

Excess light or irradiance is harmful to plants that are unable to balance the ratio of absorbed/used energy (Wagner et al., 2006). Once photochemical capacity is exceeded by incoming energy and photoprotective mechanisms are overwhelmed, ROS, such as O_2_^–^, H_2_O_2_, and OH^–^, can be produced (Murchie and Ruban, 2020). The ROS then react with membrane lipids and proteins, thereby leading to photoinhibition and/or irreversible photooxidative damage (Krieger-Liszkay et al., 2008). Non-photochemical quenching (NPQ) is believed to be an effective photoprotective process, in which excess absorbed light energy is safely dissipated as heat. Both PsbS and the xanthophyll cycle are involved in non-photochemical quenching, and it is the pH gradient across the vesicle-like membrane that directly triggers NPQ (Li et al., 2000, 2004; Müller et al., 2001). The general consensus is that the protonation of PsbS occurs faster than the Vio-to-Zea conversion and that it largely determines NPQ induction/relaxation (Li et al., 2000, 2004; Horton et al., 2008). In the present study, there was an increased trend between NPQ and Φ_NPQ_ in response to simulated sunflecks, but the NPQ of monoculture system was greater than that of intercropping system (Figures 4, 5). This result indicated that the NPQ pathway in the intercropping system is gradually released to utilize more light energy for photosynthesis, and the establishment of NPQ is closely related to light intensity. This observation was consistent with a previous research in *A. thaliana*, which showed the decreased fitness of plants grown under high light due to defective *PsbS* in NPQ (Külheim et al., 2002), Thus, the elevation of NPQ in response to intense sunflecks maybe be ascribed to the presence of PsbS.

Cyclic electron flow around photosystem I (CEF) has been documented as an important mechanism of protecting PSI and PSII in plants exposed to abiotic stress (Huang et al., 2012). CEF-deficient mutants of *A. thaliana* exhibited a slope in photosynthesis, accompanying with plant growth retardation (Munekage et al., 2004). The CEF protects PSII from photo-inhibition through activating NPQ and stabilizing oxygen-evolving complex (OEC) (Heber and Walker, 1992; Huang et al., 2016). Previous studies have also confirmed that *Triticum aestivum* and *Paraboea rufescens* protect PSII by activating CEF to drive thermal dissipation under environmental stress (Golding and Johnson, 2003; Huang et al., 2012). There was a rapid elevation in NPQ between monoculture leaves and intercropping leaves in response to simulated sunflecks (Figure 4F), indicating that the activation of CEF might enhance the thermal dissipation and stabilize OEC to protect the PSII. Additionally, CEF-mediated ATP synthesis also plays an important role in the repair of PSII (Allakhverdiev et al., 2005; Yamori et al., 2016). Therefore, high light stress or intense sunflecks might cause an elevation in CEF-mediated ATP synthesis, which in turn leads to a greater rate of PSII repair than of PSII photo-damage, this is in line with previous results that the rate of PSII repair was reduced upon an inhibition on the synthesis of CEF-mediated ATP in response to transient strong light (Allakhverdiev et al., 2005).

NPQ responded to simulated sunflecks very rapidly, as has been demonstrated for tropical rainforest shade-tolerant species (Logan et al., 1997; Watling et al., 1997; Tausz et al., 2005). In response to simulated sunflecks, there was a rapid increase in NPQ between monoculture leaves and intercropping leaves (Figure 4F), consistent with previous studies (Watling et al., 1997; Tausz et al., 2005). The change in NPQ is mainly due to pH-dependent quenching, which engages and reverts quickly (Maxwell and Johnson, 2000). Δ*F*/*F*_m_′ declined to near zero at the start of the simulated sunfleck (Figure 4G), which was likely related to simultaneous closure of available reaction centers of PSII. Thereafter, Δ*F*/*F*_m_′ slowly increased with the process of photosynthetic induction (Figure 4G), consistent with previous studies (Watling et al., 1997; Tausz et al., 2005). In addition, when being exposed to a simulated sunfleck, a comparable value of Φ_NPQ_ was observed in the two treated leaves but the intercropping leaves showed a higher value of Φ_PSII_ (Figures 5C,D). These results indicated that higher photosynthetic capacity would correspondingly result in a slightly smaller engagement of NPQ in the intercropping leaves, when subject to a sudden increase in PFD.

The xanthophyll cycle is known to represent one of the main photoprotection mechanisms in plant cells, which has multiple functions such as thermal dissipation, protection against oxidative stress caused by light, modulation of the structure of thylakoid membrane, blue light signal transduction, and regulation of the synthesis of abscisic acid (ABA) (Goss and Jakob, 2010; Xiong et al., 2012). At midday under field conditions, when light was the sole stress, plants protected themselves from high light stress through thermal dissipation, which dissipated up to 75% of the energy absorbed by plants leaves (Wang, 2010). In this study, a significant difference was observed in the mass-based pool size of xanthophyll cycle, i.e., (V + A + Z), between intercropping leaves and monoculture leaves (Table 2). The extent of the de-epoxidation of the pigment interconversion within the xanthophyll cycle can be described by the (Z + A)/(V + A + Z) ratio. Although the monoculture leaves exhibited a larger mass-based pool size of xanthophyll cycle than the intercropping leaves, the intercropping leaves had a higher ratio of (Z + A)/(V + A + Z) than the monoculture leaves (Table 2), which could to some extent compensate for the shortfalls in the xanthophyll cycle pool size of the intercropping leaves and thus help them to protect themselves from injury by intense sunflecks. On the other hand, the xanthophyll cycle pigments Z and A are formed from V when plants are exposed to excess light. In fact, some previous studies have demonstrated that the xanthophyll cycle pigment-Z could quench the singlet state oxygen (Demmig-Adams and Adams, 2006; Havaux et al., 2007), and thus the xanthophyll cycle could also protect lipids from oxidative stress caused by excess absorbed light. Therefore, the xanthophyll cycle plays a vital role in the photoprotection of intercropping leaves and monoculture leaves of *A. xiei* by dissipating more excess energy when exposed to high light stress or intense sunflecks.

β-car is an important auxiliary pigment in plant cells. High content of β-car could dissipate excess excitation energy and scavenge reactive oxygen species, protecting photosynthetic apparatus from damage (Ngwene et al., 2017). Mutants perturbed in the accumulation of lycopene cyclase are sensitive to high light, as evidenced by β-car loss and lower qP (Cazzaniga et al., 2012). In the present study, β-car content in the intercropping system was increased approximately twofold compared to that of the monoculture system (Table 2). A reasonable explanation could be that β-car is not comprised of N atoms and their accumulation is beneficial for absorption of light energy to be served as photosynthesis, thereby protecting Chl from photo-damage (Ruban et al., 2004). Likewise, the expression of genes involved in β-car biosynthesis were shown to be up-regulated in *Haematococcus pluvialis* grown under high light condition (Grünewald et al., 2000). Thus, β-car accumulation might prevent over reduction of the photosynthetic electron transport chain and the formation of reactive oxygen species by intense sunflecks.

During normal metabolism, ROS are generated as a side product in the photosynthetic transport of electrons, such as photosynthesis and respiration. Once the balance between the production of ROS and the quenching activity of antioxidants becomes upset, plants will be subjected to oxidative stress, often resulting in oxidative damage (Hare et al., 1998; Taylor et al., 2004). Under conditions of intense illumination or sunflecks, the imbalance between light absorption and the ultilization of photosynthetic light or sunflecks might occur, which can potentially result in the production of ROS. Thus, photoprotection is needed when plants are exposed to high-light stress or intense sunflecks. The ascorbate-glutathione cycle is a metabolic pathway that detoxifies H_2_O_2_, and plays a critical role in keeping ROS under control in plants (Noctor and Foyer, 1998). In this cycle, four antioxidant enzymes (APX, GR, DHAR, and MDHAR) are involved in the removal of H_2_O_2_ and of O_2_^–^ radicals. In the present study, oxygen free radicals were more likely to accumulate in the monoculture leaves than in the intercropping leaves (Table 3). On the other hand, the greater increase in glutathione content (Table 3) could help to quench ROS, thereby resulting in the less accumulation of oxygen free radicals in the intercropping leaves.

## Conclusion

The intercropping leaves of *A. xiei* demonstrate a very fast response to sunflecks, and this can help them maintain a high positive carbon gain, because of their high rates of photosynthesis compared with the monoculture counterparts; this is thus highly advantageous to its final yield under shade conditions of the intercropping system. The intercropping leaves of *A. xiei* apparently retain robust photoprotective mechanisms such as xanthophyll cycle-dependent energy dissipation and the enzymatic antioxidant defense system, which are comparable to those of their monoculture counterparts. Taking the low-light environment inhabited by the intercropping leaves of *A. xiei* into account, these large biochemical investments in photoprotection might represent a wasteful use of resources. However, these investments could efficiently protect the leaves from the injury from intense sunflecks, alleviate photoinhibition, and thus might be associated with the conservative behavior linked to the origin of this species as a shade-tolerant plant.

## Data Availability Statement

The original contributions presented in the study are included in the article/supplementary material, further inquiries can be directed to the corresponding author/s.

## Author Contributions

JZ wrote the manuscript and made a statistical analysis of the experimental data. JC and SX designed the experiment and provided ideas for writing manuscript. SS and LZ were responsible for the measurement of steady-state gas exchange and photosynthetic induction during simulated sunflecks. All authors agreed to the publication of this article.

## Conflict of Interest

The authors declare that the research was conducted in the absence of any commercial or financial relationships that could be construed as a potential conflict of interest.

## Abbreviations

A: antheraxanthin
APX: Ascorbate peroxidase
AQY: apparent quantum yield
CAT: Catalase
Ci: intercellular CO_2_ concentration
DHAR: Dehydroascorbate reductase
ETR: electron transport rate
Fv′/Fm′: maximum efficiency of PSII photochemistry in the light
Δ *F/F*m′: actual efficiency of PSII photochemistry in the light
Φ f,d: combined quantum efficiency of fluorescence and constitutive thermal dissipation
LCP: light compensation point
LSP: light saturation point
Rd: dark respiration rate
t50%: time required to reach 50% of Pmax
PFD: photon flux density
Pmax: maximum photosynthetic assimilation rate
PB: the amount of nitrogen partitioned to bioenergetics
PL: the amount of nitrogen partitioned to light harvesting
POD: Peroxidase
PC: the amount of nitrogen partitioned to carboxylation
Φ PSII: quantum efficiency of photochemical transports
MDA: Malondialdehyde
MDAR: Monodehydroascorbate
NPQ: non-photochemical quenching
Φ NPQ: quantum efficiency of light-dependent thermal dissipation
ROS: Reactive oxygen species
SLA: specific leaf area
SOD: Superoxide dismutase
t90%: time required to reach 90% of Pmax
V: violaxanthin
Z: zeaxanthin.
